# Xyla-P Cream vs. Lidocaine Spray: Impact on Patient Satisfaction, Anxiety, Cooperation, and Pain in Spinal Anesthesia for Cesarean Section

**DOI:** 10.5812/aapm-157126

**Published:** 2025-01-12

**Authors:** Zahra Ghalenoii, Shohreh Movahedi, Amirabbas Motezaker, Elham Ebrahimi

**Affiliations:** 1 Department of Obstetrics and Gynecology, Baharlou Hospital, School of Medicine, Tehran University of Medical Sciences, Tehran, Iran; 2 Nursing and Midwifery Care Research Center, Department of Reproductive Health Midwifery, School of Nursing & Midwifery, Tehran University of Medical Sciences, Tehran, Iran

**Keywords:** Cesarean Section, Spinal, Lidocaine, Xyla-P

## Abstract

**Background:**

One of the fundamental principles of medical interventions is to avoid causing pain to patients, and childbirth is no exception. With the rising prevalence of cesarean sections, addressing factors that may diminish maternal satisfaction is crucial. Spinal anesthesia, the most common method for cesarean sections, faces challenges such as patient anxiety. To mitigate pain associated with needle insertion, various methods, including lidocaine spray and Xyla-P cream, have been recommended.

**Objectives:**

This study aimed to evaluate the effectiveness of lidocaine spray and Xyla-P cream in reducing pain during needle insertion for spinal anesthesia in cesarean sections.

**Methods:**

This randomized, placebo-controlled interventional study included 263 pregnant women at 37 weeks or more of gestational age who were candidates for elective cesarean sections. Participants were randomly assigned to intervention and control groups using a block permutation technique. In intervention group 1, 10 g of Xyla-P cream was applied 30 minutes before spinal anesthesia. In intervention group 2, three puffs of 10% lidocaine spray were used. The control group received three puffs of water spray ten minutes before anesthesia. Pain intensity and anxiety were assessed using the Visual Analog Scale (VAS), and maternal cooperation was scored by the anesthesiologist.

**Results:**

The mean age of the participants was 30 years, and 21% had no prior history of cesarean section. There was no significant difference in pain, anxiety, satisfaction, and cooperation between the Xyla-P and lidocaine groups. However, in the group receiving lidocaine, satisfaction (P-value: 0.001) and cooperation (P-value: 0.019) improved significantly compared to the placebo group, whereas anxiety increased significantly compared to the placebo group (P-value: 0.045).

**Conclusions:**

Lidocaine had a positive effect on maternal satisfaction with spinal anesthesia and, compared to the placebo, led to significant improvements in maternal satisfaction and cooperation. In light of these findings, lidocaine emerges as a more appropriate choice than Xyla-P cream.

## 1. Background

Cesarean section is a surgical procedure, and Iran and Turkey have the highest rates at 47%, with some local studies reporting rates exceeding 50% (1, 2). During the COVID-19 pandemic, the frequency of cesarean births increased significantly, rising from 50.8% to 52.9% of all births, which is notably higher than the rate in countries like the United States (30%) (3). 

One of the fundamental principles of all medical interventions is to minimize pain or discomfort to patients, and childbirth is no exception. Procedural discomfort has been identified as a critical factor in improving patient outcomes and satisfaction, but it is often overlooked for various reasons (4). With the increasing trend toward cesarean births, particularly in high-risk cases, attention to factors that contribute to maternal dissatisfaction is essential. Severe acute pain during cesarean section has been identified as a primary factor reducing maternal satisfaction and causing post-traumatic stress disorder (PTSD)(5). 

Spinal anesthesia is the most common anesthesia method for cesarean sections, used in 93% of cases (6-8). A 2012 study in Gonabad, Iran, reported that 48% of pregnant women undergoing cesarean section chose spinal anesthesia, while a similar study in Tehran found that 75% of mothers opted for it, with maternal age directly related to the preference for elective cesarean sections (9). Local anesthesia methods have been associated with moderate satisfaction among pregnant women, and spinal anesthesia is generally preferred over general anesthesia due to its lower risk profile. 

A significant challenge for anesthesiologists during spinal anesthesia is managing patients' anxiety and fear of the pain caused by needle insertion. These factors can lead to patient non-cooperation, increasing the likelihood of transitioning to general anesthesia, which carries risks such as prolonged anesthesia time, aspiration, post-operative nausea and vomiting, and airway damage (10). Local anesthesia has been identified as a key factor in reducing anesthesia failure and improving patient positioning during needle insertion (11). 

Various methods have been proposed for skin anesthesia in painful procedures like spinal anesthesia and venous cannulation. These include skin patches, creams, and lidocaine sprays. Studies have shown that local anesthesia effectively reduces pain during venous cannulation without significantly increasing failure rates (12). The use of 10% lidocaine spray offers advantages such as ease of application and a shorter onset time for skin anesthesia. Topical lidocaine has also been effective in reducing pain during hysteroscopy and hysterosalpingography (8, 13). 

The discomfort associated with pre-epidural lidocaine injections has led to a preference for alternative formulations like sprays and creams. However, lidocaine spray may have limitations, such as delayed onset of anesthesia and reduced efficacy if the procedure is prolonged. Combined anesthetic creams, like Xyla-P cream, offer an alternative. Xyla-P cream contains 5% lidocaine and 2% prilocaine, providing adequate anesthesia with minimal invasiveness and toxicity risk. Its efficacy in reducing procedural pain has been demonstrated, including in studies on EMLA cream (lidocaine-prilocaine) (14). 

## 2. Objectives

The primary objective of this study was to assess patient satisfaction when using 10% lidocaine spray versus Xyla-P cream before cesarean sections. This satisfaction was evaluated in relation to pain, anxiety, and patient cooperation. By analyzing these parameters, this research compared the effects of Xyla-P cream and lidocaine spray on patient satisfaction during spinal anesthesia for cesarean sections. 

## 3. Methods

This interventional study was a single-blind randomized clinical trial conducted at Baharloo Hospital, Tehran, Iran, with approval from the Ethics Committee of Tehran University of Medical Sciences (Ref. ID: IR.TUMS.MEDICINE.REC.1401.702). A total of 263 pregnant women with a gestational age of over 37 weeks who were candidates for elective cesarean section participated in this study. Exclusion criteria included gestational age less than 37 weeks, emergency cesarean sections, chronic conditions such as hypertension and diabetes, a history of allergy to lidocaine, a history of addiction, and contraindications for spinal anesthesia. All participants were informed about the study procedures, potential complications, and risks, and they provided written informed consent. 

After receiving an explanation of the study procedures and being informed that they would not know their group assignment, the mothers were randomly assigned to two groups of 86 and one group of 91, totaling 263 participants, using block randomization with a block size of four. In intervention group 1, 10 g of Xyla-P cream (manufactured by Tehran Chemistry Pharmaceutical Company) was applied to a 10 cm² surface area (as determined by the anesthesiologist) 20 - 30 minutes before spinal anesthesia at the needle insertion site to induce local anesthesia. A patch was then applied, and the mother underwent spinal anesthesia using a G-25 needle and 0.5% marcaine ampoule (manufactured by Aspen Company). 

In intervention group 2, 3 puffs of 10% lidocaine spray (manufactured by Doniaiebehdasht Company) were applied to the needle insertion site 5 - 10 minutes before spinal anesthesia. In the control group, 3 puffs of distilled water were used 10 minutes before spinal anesthesia. 

To comprehensively assess the patient experience, demographic data were collected, and evaluations included pain intensity measured using the Visual Analog Scale (VAS), anxiety levels assessed according to the research of Lesage et al., and the anesthesiologist's evaluation of maternal cooperation (15-17).

The VAS was used to measure pain, with patients rating their pain on a scale of 0 to 10. Pain intensity was categorized as follows: Mild (0 - 2), moderate (3 - 7), and severe (8 - 9). Additionally, pain could also be assessed by an observer based on the patient's facial expressions, with 0 indicating no pain, 1 indicating mild pain, 2 indicating more than mild pain, 3 indicating moderate pain, 4 indicating severe pain, and 5 indicating very severe pain.

Anxiety levels were also assessed using the VAS, categorized as follows: Mildly distressing (0 - 2), uncomfortable (2 - 4), painful (4 - 6), terrifying (6 - 8), and unbearable (8 - 10).

Cooperation was evaluated based on the patient’s compliance with treatment protocols and active participation in assessments. A researcher-developed checklist was utilized to specifically measure patient satisfaction related to the three different anesthesia methods (lidocaine spray, distilled water, and lidocaine ointment). This checklist included comprehensive questions aimed at evaluating various aspects of the patients' experiences. The questions addressed factors such as the quality of pain control, side effects, and the patients’ understanding of the process and information provided. Responses were rated on a 1 to 5 scale, allowing for quantifiable analysis of satisfaction.

### 3.1. Statistical Analysis

Data collected from the forms were entered into SPSS version 19. For the descriptive analysis of quantitative variables, mean and standard deviation were utilized, while frequency and percentage were employed for qualitative variables. One-way analysis of variance (ANOVA) was conducted to compare the means among the three groups. Pairwise comparisons between groups were performed using the Least Significant Difference (LSD) test. The chi-square test was applied to compare qualitative variables. A P-value of less than 0.05 was considered statistically significant.

## 4. Results

**Figure 1.**
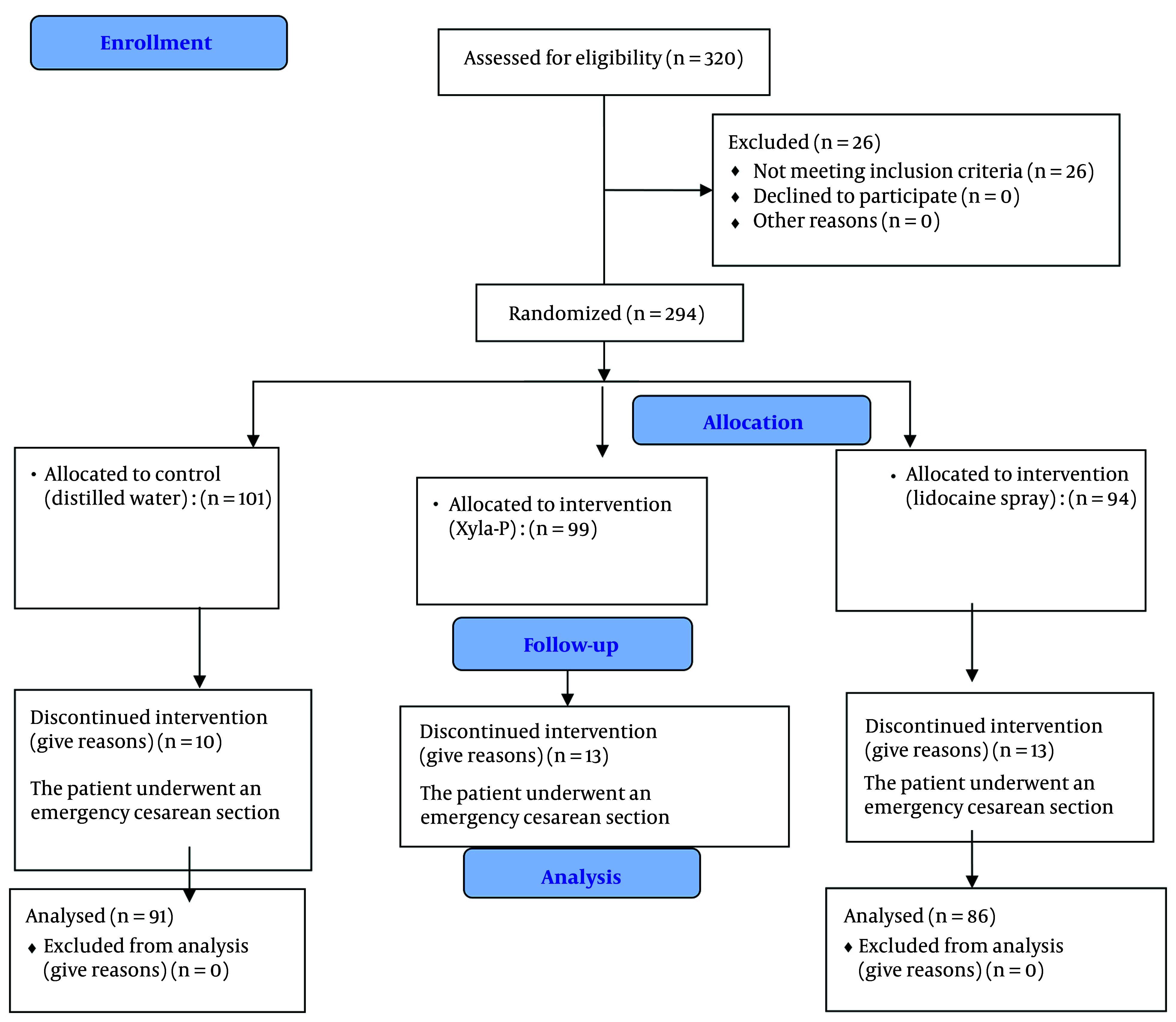
CONSORT flow diagram of participants

A total of 263 pregnant women over 37 weeks of gestation who met the inclusion criteria were included in the study. A CONSORT flow diagram of the participants is presented in Figure 1. The mean age of the participants was 30 years, and the average BMI was 30.3 (Figure 1). 

Among all women participating in this study, 69% had no underlying diseases, 21% had no previous history of cesarean section, 78.2% had prior cesarean experiences, and 4.3% were smokers. There was a significant statistical difference between the study groups in terms of smoking and BMI.

Additionally, the number of miscarriages, live births, reasons for cesarean sections, history of diseases, medications used, and history of cesarean surgery showed significant differences between the groups. Other demographic information and maternal pregnancy-related details are listed separately for the intervention groups in Table 1. 

**Table 1.** Pregnancy and Delivery Variables ^a^

Variables	Total (N = 263)	Xyla-P (n = 86)	Lidocaine (n = 86)	Placebo (n = 91)	P-Value
**Gravidity**	2.5 ± 1.07	2.5 ± 1.19	2.6 ± 1.19	2.5 ± 0.80	0.867
**Number of live births**	1.2 ± 0.83	1.1 ± 0.82	1.2 ± 0.88	1.4 ± 0.76	0.026
**Number of miscarriage**	0.3 ± 0.65	0.4 ± 0.72	0.4 ± 0.77	0.1 ± 0.35	0.005
**Gestational age**	38.3 ± 0.80	38.3 ± 0.86	38.3 ± 0.71	38.3 ± 0.83	0.849
**Indications for C-section**					< 0.001
Elective	48 (18.5)	27 (31.4)	21 (24.4)	0	
Breech presentation and fetal anomalies	4 (15)	0	0	4 (4.6)	
Repeat C-section	201 (77.6)	59 (68.6)	65 (75.6)	77 (88.5)	
Large for gestational age	4 (15)	0	0	4 (4.6)	
**Disease history**					0.019
None	180 (69.0)	49 (57.0)	64 (74.4)	67 (75.3)	
Diabetes	19 (7.1)	6 (7.0)	4 (4.7)	9 (10.1)	
Thyroid problems	25 (9.4)	13 (15.1)	7 (8.1)	5 (5.6)	
Hypertension	4 (15)	0	1 (1.2)	3 (3.4)	
Two or more diseases	13 (5.0)	8 (9.3)	3 (3.5)	2 (2.2)	
Other	22 (8.0)	10 (13.6)	7 (9.1)	5 (3.4)	
**Drug history**					0.022
None	190 (73.6)	50 (59.5)	68 (79.1)	72 (79.0)	
Levothyroxine	27 (10.5)	13 (15.5)	9 (10.5)	5 (5.4)	
Insulin or metformin	15 (5.8)	6 (7.2)	2 (2.4)	7 (7.9)	
Two or more	11 (4.3)	7 (8.3)	2 (2.3)	2 (2.3)	
Other	20 (5.8)	10 (9.5)	5 (5.7)	5 (5.4)	
**Surgery history**					0.013
None	57 (21.8)	27 (31.4)	20 (23.3)	10 (11.2)	
C-section	204 (78.2)	59 (68.6)	66 (76.7)	79 (88.8)	
**Number of attempts for epidural anesthesia**	1.2 ± 0.61	1.3 ± 0.64	1.2 ± 0.45	1.3 ± 0.73	0.668

^a^ Values are expressed as mean ± SD or No. (%).

The mean levels of pain, anxiety, satisfaction, and cooperation were compared among the study groups. Satisfaction levels among pregnant women in the Lidocaine spray group were higher than those in the Xyla-P cream group, and both were higher than those in the placebo group, with this difference being statistically significant (P-value = 0.003). The mean pain level in the Lidocaine group was lower compared to the Xyla-P cream and placebo groups, although the difference was not significant. Anxiety levels were lower in the placebo group compared to the other two groups, but this difference was also not statistically significant. Cooperation levels decreased progressively from the Lidocaine group to the Xyla-P cream group and then the placebo group, but these differences were not significant.

Patient satisfaction in the Xyla-P cream group showed a significant increase compared to the placebo group (P-value = 0.008). However, for other parameters, including anxiety, pain, and cooperation, the differences between the Xyla-P cream and placebo groups were not statistically significant. Additionally, there was no significant difference in pain, anxiety, satisfaction, and cooperation levels between the Xyla-P cream and Lidocaine groups.

In the Lidocaine group, satisfaction (P-value = 0.001) and cooperation (P-value = 0.019) showed significant improvement compared to the placebo group. However, anxiety levels increased significantly in the Lidocaine group compared to the placebo group (P-value = 0.045). (Table 2) 

**Table 2.** Comparison of Average Scores for Pain, Stress, Anxiety, Satisfaction, and Cooperation Among Study Groups ^a^

Variables	Total (N = 263)	Xyla-P (n = 86)	Lidocaine Spray (n = 86)	Placebo (n = 91)	P-Value	Post-hoc
**Pain**	3.5 ± 2.55	3.6 ± 2.76	3.2 ± 2.12	3.5 ± 2.68	0.539	Placebo vs Lidocaine: P = 0.06 <br> Placebo vs Xyla-P: P = 0.07 <br> Lidocaine vs Xyla-P: P = 0.75
**Stress and anxiety**	6.3 ± 3.31	6.4 ± 3.33	6.8 ± 3.22	5.7 ± 3.34	0.127	Placebo vs Lidocaine: P = 0.07 <br> Placebo vs Xyla-P: P = 0.06 <br> Lidocaine vs Xyla-P: P = 0.85
**Satisfaction**	8.5 ± 1.96	8.7 ± 1.84	8.9 ± 1.57	7.9 ± 2.26	0.003	In all cases P = 0.000
**Cooperation**	8.4 ± 1.90	8.4 ± 2.10	8.8 ± 1.34	8.1 ± 2.12	0.064	Placebo vs Lidocaine: P = 0.06 <br> Placebo vs Xyla-P: P = 0.06 <br> Lidocaine vs Xyla-P: P = 0.62

^a^ Values are expressed as mean ± SD.

## 5. Discussion

In this study, we compared the average levels of pain, anxiety, satisfaction, and cooperation among three groups of pregnant women receiving different anesthesia methods. Our findings revealed that satisfaction levels in the lidocaine spray group were significantly higher than those in the Xyla-P cream group, with both groups reporting greater satisfaction than the placebo group. However, no significant differences were observed in the mean levels of pain, anxiety, and cooperation among the study groups. Notably, both Xyla-P cream and lidocaine spray were significantly associated with improved satisfaction compared to the placebo.

Regional anesthesia, including spinal and epidural techniques, offers numerous advantages during cesarean sections. This approach not only provides effective pain relief during labor but also allows mothers to remain awake and alert throughout the procedure (18). This is especially important for mothers who wish to experience the birth of their child or for those for whom general anesthesia poses additional risks. A review by Chohan et al. highlighted regional anesthesia, particularly the epidural method, as the preferred choice for pain relief in mothers with underlying heart conditions, such as atrial or ventricular septal defects, emphasizing the importance of effective pain management during all stages of this anesthetic approach (19).

Additionally, a study conducted in 2002 involving 1,619 pregnant women reported that the length of postpartum hospitalization was 3 - 4 days shorter for those receiving spinal anesthesia compared to those undergoing general anesthesia. Spinal anesthesia has a faster onset, shorter recovery time, and reduces the risk of postoperative complications such as nausea, vomiting, respiratory disorders, and fetal complications (20). Djabatey and Barclay compared mortality rates between spinal and general anesthesia, reporting rates of 6.5 and 3.8 per million, respectively (21). It is important to note, however, that patients undergoing general anesthesia may face higher risks associated with emergency labor and specific conditions, such as placental adhesion (22).

Lidocaine is the most commonly used topical anesthetic in cesarean sections, with its efficacy demonstrated in various diagnostic and therapeutic procedures (23). Given that the injection of lidocaine prior to epidural needle insertion can cause procedural discomfort, there is growing interest in alternative formulations such as sprays or ointments. A systematic review by Fettes et al. identified topical anesthesia as a factor that reduces the failure of spinal anesthesia and assists in maintaining proper patient positioning during needle insertion (24).

Long-term complications of cesarean sections, including chronic postpartum pain, are often linked to the mother's experience during childbirth. A review of 63 studies by Weinstein et al. concluded that the incidence of chronic post-surgical pain is lower with regional anesthesia compared to general anesthesia (25). Similarly, findings from Vermelis et al. indicated that general anesthesia and factors like pain tolerance during cesarean sections could predict a mother's likelihood of experiencing chronic postpartum pain (26). Kita et al. further reported that severe acute pain during cesarean delivery significantly reduces maternal satisfaction and can lead to PTSD (27).

A 2015 study by Ghanaee et al., involving 100 pregnant women undergoing cesarean sections, found that topical lidocaine significantly reduced postoperative pain at the incision site by reversibly inhibiting voltage-gated sodium channels (28). They utilized the Visual Analogue Scale to assess patients' pain scores, confirming the importance of adequate anesthesia in managing pain during cesarean sections, a critical factor influencing a woman's childbirth experience. Given the increasing prevalence of cesarean sections in high- and middle-income countries, minimizing anxiety and pain for pregnant women during all stages of labor is essential. Such measures can help prevent psychological issues, including PTSD, and promote healthy mother-baby bonding.

While our study aligned with previous research in finding no significant differences in self-reported anxiety or pain between the lidocaine and Xyla-P cream groups, it further supports the efficacy of lidocaine during childbirth. Although no significant difference was noted in anxiety levels between the lidocaine and Xyla-P cream groups, lidocaine was associated with increased anxiety compared to the placebo. Overall, considering that lidocaine had a more favorable impact on maternal satisfaction compared to Xyla-P cream, and given its significant association with improved satisfaction and cooperation relative to the placebo, it appears that lidocaine is the preferable option over Xyla-P cream.

This study's limitations include a single-blind design, reliance on subjective pain and anxiety assessments, and a potentially unrepresentative sample. To mitigate biases, a double-blind design could be implemented, along with the inclusion of objective measures of physiological responses. Standardizing the timing of anesthetic application and conducting long-term follow-up assessments would enhance the reliability of pain management comparisons. Additionally, controlling for concomitant medications and collecting data on maternal factors to reduce confounding variables could improve the study's robustness. Addressing the placebo effect could further enhance study validity.

## Data Availability

The dataset presented in the study is available on request from the corresponding author during submission or after publication.

## Clinical Trial Registration Code:

IRCT20230128057251N1.
